# Self-incremental learning vector quantization with human cognitive biases

**DOI:** 10.1038/s41598-021-83182-4

**Published:** 2021-02-16

**Authors:** Nobuhito Manome, Shuji Shinohara, Tatsuji Takahashi, Yu Chen, Ung-il Chung

**Affiliations:** 1 Graduate School of Engineering, The University of Tokyo, 7-3-1 Hongo, Bunkyo-ku, Tokyo, 113-8656 Japan; 2 Graduate School of Science and Engineering, Tokyo Denki University, Saitama, Japan; 3 Graduate School of Frontier Sciences, The University of Tokyo, Chiba, Japan; 4 Graduate School of Medicine, The University of Tokyo, Tokyo, Japan

**Keywords:** Cognitive neuroscience, Learning algorithms, Computer science, Software

## Abstract

Human beings have adaptively rational cognitive biases for efficiently acquiring concepts from small-sized datasets. With such inductive biases, humans can generalize concepts by learning a small number of samples. By incorporating human cognitive biases into learning vector quantization (LVQ), a prototype-based online machine learning method, we developed self-incremental LVQ (SILVQ) methods that can be easily interpreted. We first describe a method to automatically adjust the learning rate that incorporates human cognitive biases. Second, SILVQ, which self-increases the prototypes based on the method for automatically adjusting the learning rate, is described. The performance levels of the proposed methods are evaluated in experiments employing four real and two artificial datasets. Compared with the original learning vector quantization algorithms, our methods not only effectively remove the need for parameter tuning, but also achieve higher accuracy from learning small numbers of instances. In the cases of larger numbers of instances, SILVQ can still achieve an accuracy that is equal to or better than those of existing representative LVQ algorithms. Furthermore, SILVQ can learn linearly inseparable conceptual structures with the required and sufficient number of prototypes without overfitting.

## Introduction

Despite significant advances in machine learning, the learning abilities of human beings are still much better than those of machines. For example, in many cases, humans can generalize concepts from small numbers of samples^1,2^, and even children can make meaningful generalizations with a single learning session^3–5^. On the other hand, standard algorithms in machine learning require large numbers of samples and multiple learning sessions to perform similar tasks compared with humans^6^.

However, humans are known to not always make logical inferences in daily life^7^, a tendency that is referred to as cognitive bias. Cognitive bias can sometimes lead to false inferences but is often a useful heuristic for quick cognition and decision making^8–10^.

From among a wide variety of human cognitive biases^8,9,11,12^, symmetric bias^13–15^ and mutually exclusive bias^16–18^ were focused upon in this study. Symmetric bias is a tendency to infer “if *Q*, then *P*” after being convinced of “if *P*, then *Q*,” whereas mutually exclusive bias is a tendency to infer “if not *P*, then not *Q*” after being convinced of “if *P*, then *Q*”. These inferences are illogical, but commonplace, and are thought to promote our faster learning and decision making^14–17,19–22^. These biases have also been used in the bandit problem, one of the classic reinforcement learning problems, and with naïve Bayes, a supervised learning algorithm, which have resulted in faster learning^23,24^.

In this paper, a self-incremental learning vector quantization (SILVQ) method is proposed. This method can learn concepts while autonomously adjusting the learning rate by incorporating symmetric bias and mutually exclusive bias into learning vector quantization (LVQ)^25^, a prototype-based online machine learning method. The proposed SILVQ not only has the same characteristics as those of the original LVQ, which will be described later, but also provides a learning algorithm that can be intuitively understood. This research aims to contribute to both the computer science and cognitive science fields and hopes to support the research of explainable artificial intelligence to address the black box problem of machine learning^26–28^.

The structure of this paper is as follows. First, we introduce two prominent models that can express the process of concept acquisition and explain why we used LVQ in the related works. Next, the original LVQ is reviewed. Causal induction models incorporating symmetric bias and mutually exclusive bias will then be described. Afterward, a method for automatic adjustment of learning rate, incorporating these causal induction models, will be introduced. The proposed SILVQ, which self-increases LVQ prototypes based on the aforementioned method, will then be explained. Finally, the performance of the proposed method is tested in three experiments using four real datasets (Glass^29^, Iris^30^, Ionosphere^31^, and Sonar^32^) and two artificial datasets.

## Related works

In the field of cognitive science, the formation of concepts by humans is an actively discussed topic. This section introduces the prototype-based model (PBM) and exemplar-based model (EBM), which are well-known concept formation models.

PBM abstracts the characteristics of a category from several instances belonging to a category and stores it as a prototype^33–35^. The objective of PBM is to simplify recognition and enable faster and more efficient processing of vast amounts of information. In contrast, EBM directly stores individual instances as knowledge^36–38^. In other words, the concept of PBM is abstracted knowledge, while that of EBM is the memory of individual knowledge.

We are always surrounded by vast amounts of information. Considering our storage capacity, we only need to acquire the knowledge that can be effectively used in the future from the myriad of information and conceptualize it. Therefore, our cognitive function should be modeled as a PBM. However, the limitations of PBM have been identified in some conceptual learning tasks. First, PBM can describe the typical characteristics of a category by referring to the prototype, but it cannot describe the diversity of the category. For example, the typical characteristics of the category “apple” can be described, but the range of “color,” which is a characteristic of apples, cannot be described. Meanwhile, EBM possesses a significant amount of knowledge in comparison to PBM, which has only one prototype for one category; furthermore, the diversity of categories can be described by referring to this knowledge. Second, humans can accurately reason the correlation between categories^39,40^ but PBM cannot. However, EBM can infer the correlations between categories in the same manner as the diversity of categories by referring to its knowledge. In addition, PBM can only learn linearly separable conceptual structures owing to its simple expression, while EBM can learn nonlinearly separable conceptual structures by referring to individual knowledge. Consequently, EBM has been demonstrated to be superior in various conceptual learning tasks^38,41,42^. Moreover, there is evidence that in more complex nonlinear task environments, humans refer to their own memory to perform tasks effectively^43–45^.

Although some limitations of PBM have been identified, as mentioned above, higher-order human cognitive processes often contain highly abstracted categorical information^46,47^. It is also evident that cognitive biases are responsible for the rapid and efficient cognitive processing in humans, as mentioned in the introduction. Therefore, by focusing on the cognitive biases and LVQ, which is a PBM in the field of machine learning, we have developed a machine learning model that can solve some problems related to PBM. Our model provides easy-to-interpret learning algorithms, which is one of its advantages and constitutes the main contribution of this study to the field of machine learning.

## Methods

### Learning vector quantization

LVQ is a prototype-based supervised classification algorithm that is widely used for practical classification problems because of its very simple implementation^48,49^. In addition, LVQ not only provides example-based explanations using prototypes, but also makes direct interpretation easy because the prototypes are defined in the same space as that of data^48,49^.

Among the original LVQs, LVQ1, which requires a small number of parameters to be set and has a simple learning algorithm, is described in this subsection. The purpose of LVQ is to learn a prototype for assigning an arbitrary input vector to a target class label from training data composed of an input vector $\mathbf{x}$ and a corresponding label $L\left(\mathbf{x}\right)$. Assuming that the prototype number is $i$, each prototype is composed of a prototype vector ${\mathbf{m}}_{i}$, which has the same number of attributes as that of the input vector, and a corresponding label $L\left({\mathbf{m}}_{i}\right)$. At least one prototype is prepared for each label. The LVQ1 learning algorithm is as follows.

*Step 0*. Initial values are given to the prototype vector ${\mathbf{m}}_{i}\left({0}\right)$, label $L\left({\mathbf{m}}_{i}\right)$, initial learning rate ${\alpha }_{0}$, and maximum number of learning times $T$. Furthermore, the number of learning times is set to $t={0}$.

*Step 1*. The input vector $\mathbf{x}$ and label $L\left(\mathbf{x}\right)$ are acquired as training data.

*Step 2*. The prototype $j$ closest to the input vector $\mathbf{x}$ is determined using Eq. (1).

1 $$j={\text{argmin}}_{i}\Vert \mathbf{x}-{\mathbf{m}}_{i}\left(t\right)\Vert$$

*Step 3*. The learning rate is updated using Eq. (2).

2 $${\alpha }_{j}\left(t\right)={\alpha }_{0}\left({1}-\frac{t}{T}\right)$$

*Step 4*. The prototype vector is updated using Eqs. (3) and (4).

3 $${\mathbf{m}}_{j}\left(t+{1}\right)={\mathbf{m}}_{j}\left(t\right)+s\left(t\right){\alpha }_{j}\left(t\right)\left\{\mathbf{x}-{\mathbf{m}}_{j}\left(t\right)\right\}$$

4 $$s\left(t\right)=\left\{\begin{array}{*{20}c}{1}&L\left({\mathbf{m}}_{j}\right)=L\left(\mathbf{x}\right)\\ -{1}& L\left({\mathbf{m}}_{j}\right)\ne L\left(\mathbf{x}\right)\end{array}\right.$$

*Step 5*. The number of learning times is set to $t=t+{1}$, and it returns to Step 1.

The prediction of the label for an arbitrary input vector $\mathbf{x}$ is performed by outputting the label $L\left({\mathbf{m}}_{j}\right)$ of the prototype $j$ calculated using Eq. (1). This implies that LVQ includes a prediction phase in the learning process (Step 2), in which learning is performed based on the result of “whether the label of the training data was correctly predicted.” The learning mechanism is simple: if the prediction is correct, move the prototype closer to the training data; if the prediction is incorrect, move the prototype away from the training data (Step 4).

In LVQ1, it is necessary to first set the number of prototypes per label, and the two parameters ${\alpha }_{0}$ and $T$ for determining the learning rate. Optimized LVQ1 (OLVQ1) has also been proposed as a model that improves the convergence of LVQ1 learning^25^. Furthermore, OLVQ1 does not require the setting of the parameter $T$ for determining the learning rate, unlike in LVQ1. In OLVQ1, the learning rate is updated using Eq. (5).

5 $${\alpha }_{j}\left(t\right)=\left\{\begin{array}{*{20}c}\frac{{\alpha }_{j}\left(t-1\right)}{1+s\left(t\right){\alpha }_{j}\left(t-1\right)}& t>0\\ {\alpha }_{0}&t=0\end{array}\right.$$

### Causal induction models

In the field of cognitive psychology, attempts have been made to identify how humans assess the strength of causal relationships between events^14,50,51^. Hattori describes “causal induction” as the phenomenon that induces a causal relationship between two events *P* and *Q* using their co-occurrence frequencies $a$, $b$, $c$, and $d$, as shown in Table 1^50^.

**Table 1:** Co-occurrence frequency information for event *P* and event *Q*.

	*Q*	not *Q*
*P*	$a$	$b$
not *P*	$c$	$d$

This subsection describes three causal induction models that are differentiated by the strength $R$ of the causal relationship between events based on the co-occurrence frequencies $a$, $b$, $c$, and $d$.

### CP model

Considering the conditional probability that event *Q* occurs after event *P* occurs as the strength of the causal relationship between the events, $R$ is defined as in Eq. (6).

6 $${R}^{CP}=\frac{a}{a+b}$$

This model is called a conditional probability model (CP model). The coefficient of determination between the CP model and the mean human evaluation is ${r}^{2}=\text{0.73}$^14,50,52^.

### RS model

The difference of the CP model from the mean human evaluation is considered to be due to the effect of human cognitive bias. Therefore, we define $R$ for a model incorporating symmetric bias and mutually exclusive bias, as shown in Eq. (7).

7 $${R}^{RS}=\frac{a+d}{a+b+c+d}$$

This model is called a rigidly symmetric model (RS model) because the symmetric bias and mutually exclusive bias work rigidly. The coefficient of determination between the RS model and the mean human evaluation is ${r}^{2}=\text{0.72}$^14,50,52^.

### LS model

The RS model includes a symmetric bias and a mutually exclusive bias, but these biases are unlikely to work strongly under all circumstances. Therefore, we define $R$ for a model in which symmetric bias and mutually exclusive bias are slightly effective, as demonstrated in Eq. (8).

8 $${R}^{LS}=\frac{a+\left(\frac{b}{b+d}\right)d}{a+b+\left(\frac{a}{a+c}\right)c+\left(\frac{b}{b+d}\right)d}$$

This model is called a loosely symmetric model (LS model) because the symmetric bias and mutually exclusive bias act loosely. In particular, the coefficient of determination between the LS model and mean human evaluation is confirmed to be ${r}^{2}=\text{0.91}$, which is much higher than those of the CP model and the RS model^14,52^.

### Method for automatic adjustment of learning rate

In this subsection, we describe the method for automatically adjusting the learning rate that incorporates causal induction models. This method automatically adjusts the learning rate by calculating the label confidence based on the result of “whether the label of the training data was correctly predicted” by including the prediction phase in the learning process, like in LVQ.

**Figure 1:**
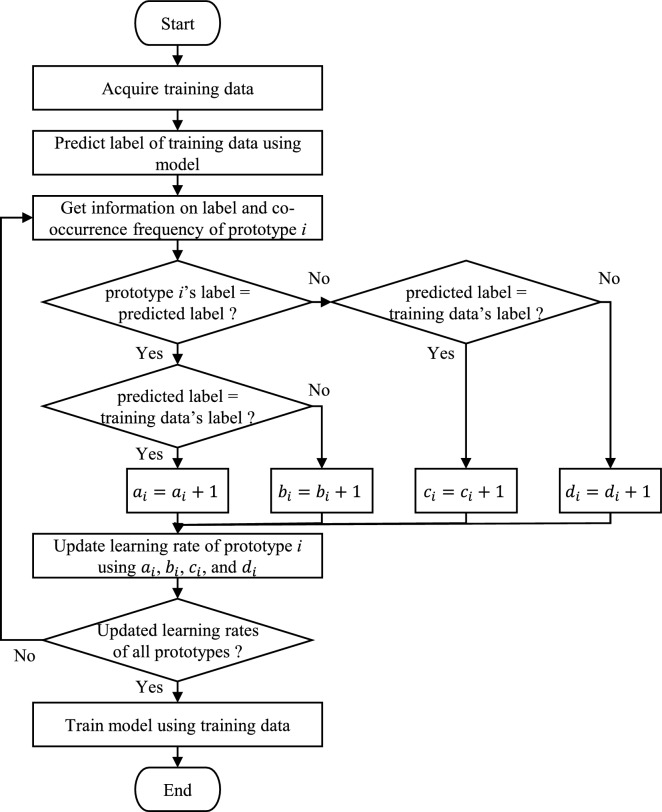
Flowchart of method for automatic adjustment of learning rate.

A method of updating the learning rate will be described using, as an example, a model having a label $L\left({\mathbf{m}}_{i}\right)$ and a learning rate ${\alpha }_{i}$ corresponding to a prototype $i$. In this method, each prototype $i$ holds the co-occurrence frequencies ${a}_{i}$, ${b}_{i}$, ${c}_{i}$, and ${d}_{i}$. This model may be applicable to all online machine learning models, but this study assumes that it is a type of LVQ. Figure 1 shows a flowchart illustrating how this method processes one instance of training data. The process of updating the learning rate after one instance of training data is acquired is as follows. First, a label $L\left(\mathbf{x}\right)$ of training data is predicted using a model, resulting in a predicted label $L\left({\mathbf{m}}_{j}\right)$ as output. Based on the prediction results, the co-occurrence frequencies ${a}_{i}$, ${b}_{i}$, ${c}_{i}$, and ${d}_{i}$ of the events outlined in Table 2 are then updated for each prototype $i$. The meanings of the two events listed in Table 2 are “the predicted label is the prototype $i$’s label” and “the predicted result is correct.” That is, the strength ${R}_{i}$ of the causal relationship between these events can be considered as the label confidence, indicating whether the prototype $i$ can correctly predict the label of the training data. With the use of ${a}_{i}$, ${b}_{i}$, ${c}_{i}$, ${d}_{i}$, and the causal induction model, ${R}_{i}$ is then calculated as the label confidence of prototype $i$. Finally, the learning rate ${\alpha }_{i}$ is updated as ${1}-{R}_{i}$. In other words, the degree of lack of label confidence is determined as the learning rate.

**Table 2:** Co-occurrence frequency information for each prototype $i$.

	$L\left( {{\mathbf{m}}_{j} } \right) = L\left( {\mathbf{x}} \right)$	$L\left( {{\mathbf{m}}_{j} } \right) \ne L\left( {\mathbf{x}} \right)$
$L\left( {{\mathbf{m}}_{i} } \right) = L\left( {{\mathbf{m}}_{j} } \right)$	$a_{i}$	$b_{i}$
$L\left( {{\mathbf{m}}_{i} } \right) \ne L\left( {{\mathbf{m}}_{j} } \right)$	$c_{i}$	$d_{i}$

### Self-incremental learning vector quantization

As described previously, because the method for automatic adjustment of learning rate includes a prediction phase in the learning process similar to in LVQ, the method can be naturally implemented in LVQ. SILVQ is different from LVQ1 and OLVQ1 such that each prototype $i$ holds the co-occurrence frequencies ${a}_{i}$, ${b}_{i}$, ${c}_{i}$, and ${d}_{i}$, and the number of prototypes per label is usually 1 at the beginning. SILVQ has a confidence threshold $\theta$ as the only parameter that needs to be set in advance. The SILVQ learning algorithm is as follows.

*Step 0*. Initial values are given to the prototype vector ${\mathbf{m}}_{i}\left({0}\right)$, the label $L\left({\mathbf{m}}_{i}\right)$, and confidence threshold $\theta$. Furthermore, the number of learning times is set to $t={0}$, and co-occurrence frequencies are set to ${a}_{i}={0}$, ${b}_{i}={0}$, ${c}_{i}={0}$, and ${d}_{i}={0}$.

*Step 1*. The input vector $\mathbf{x}$ and label $L\left(\mathbf{x}\right)$ are acquired as training data.

*Step 2*. The prototype $j$ closest to the input vector $\mathbf{x}$ is determined using Eq. (1). Furthermore, the prototype $k$ where $L\left({\mathbf{m}}_{i}\right)=L\left(\mathbf{x}\right)$ closest to the input vector $\mathbf{x}$ is determined using the same calculation as Eq. (1).

*Step 3*. For each prototype $i$, the co-occurrence frequencies ${a}_{i}$, ${b}_{i}$, ${c}_{i}$, and ${d}_{i}$ shown in Table 2 are updated, and the label confidence ${R}_{i}$ is calculated using one of the Eqs. (6)–(8). Thereafter, the learning rate is set to ${\alpha }_{i}={1}-{R}_{i}$.

*Step 4*. If $L\left({\mathbf{m}}_{j}\right)\ne L\left(\mathbf{x}\right)$ and ${R}_{j}>\theta$, the input vector $\mathbf{x}$ and the corresponding label $L\left(\mathbf{x}\right)$ are added to the model as a new prototype. If this condition is not satisfied, the prototype vector is updated Eq. (9).

9 $${\mathbf{m}}_{k}\left(t+{1}\right)={\mathbf{m}}_{k}\left(t\right)+{\alpha }_{k}\left(t\right)\left\{\mathbf{x}-{\mathbf{m}}_{k}\left(t\right)\right\}$$

*Step 5*. The number of learning times is set to $t=t+{1}$, and it returns to Step 1.

Equation (9) denotes simply updating the prototype vector that has the same label as the training data. The condition of adding a prototype indicates that the prediction is wrong even though the label confidence is higher than an arbitrary threshold. Adapting the process of a human learning knowledge, the learning mechanism of SILVQ is as follows.

- (A) Confidence is low, but the prediction is correct.
  → Knowledge is greatly modified, and confidence is raised.
- (B) Confidence is low, and the prediction is incorrect.
  → Knowledge is greatly modified, and confidence is further lowered.
- (C) Confidence is high, and the prediction is correct.
  → Knowledge is hardly modified, and confidence is further raised.
- (D) Confidence is high, but the prediction is incorrect.
  → Knowledge with new features is learned, and confidence is lowered.

This learning mechanism will be explained using, as an example, the process of a child learning the knowledge of “apple.” When a child learns “apple” for the first time, the child’s knowledge will be greatly modified because of low confidence (A & B). When a child who knows apple well learns “apple,” the child’s knowledge will hardly be modified because of the high confidence (C). However, when a child who knows apple well as a red apple learns for the first time that a green apple is also an “apple,” it is natural to learn this as knowledge with new features (D). In other words, a child who was convinced of “red” as a feature of an apple would not modify his/her knowledge to “yellow,” which is a neutral color between red and green, even if he/she saw the green apple for the first time. This method not only removes the need for setting the number of prototypes per label and the parameters for determining the learning rate, but also provides a natural learning algorithm that works by calculating the confidence of the knowledge.

## Experiments

This subsection describes three experiments performed to evaluate the performance of the proposed method. Table 3 lists information on the datasets used in this experiment.

**Table 3:** Dataset information used in experiments 1, 2, and 3.

Datasets	Instances	Attributes	Labels
Glass	214	9	6
Iris	150	4	3
Ionosphere	351	34	2
Sonar	208	60	2
Artificial dataset 1	500	2	5
Artificial dataset 2	300	2	3

### Experiment 1

In this experiment, the performance of SILVQ was verified in an environment in which each instance was learned only once, which is like what a human would encounter on a daily basis.

The experimental procedure is as follows. First, an arbitrary dataset is randomly shuffled. The dataset is then divided into 80% training data and 20% test data. Thereafter, a model is trained by acquiring instances one by one from the training data, and accuracy is calculated in each training stage using test data. The reason why this experimental procedure is used instead of the cross-validation often performed in the performance evaluation of the machine learning model is that the performance of the model largely depends on the training order.

Four real datasets, namely, Glass, Iris, Ionosphere, and Sonar, were used to validate the performance of the model. Glass and Iris are multi-label datasets with human-understandable attributes. Ionosphere and Sonar are waveform datasets with attributes that are difficult for humans to intuitively understand. These datasets are available in the UCI Machine Learning Repository^53^, which is a database collected by the machine learning community for the analysis of machine learning algorithms. The models used were SILVQ using CP, RS, and LS models as causal induction models (SILVQ–CP, SILVQ–RS, SILVQ–LS) with $\theta =\text{1.0}$, and LVQ1 and OLVQ1 with initial learning rates ${\alpha }_{0}$ of 0.5, 0.3, and 0.1. SILVQ with $\theta =\text{1.0}$ is a special form in which the number of prototypes does not increase. That is, a comparison of these models signifies a comparison of simple models that move one prototype based on the learning rate. The initial value of the prototype vector of each model was set as the vector value of an instance having a label obtained for the first time from the training data. For each dataset, the maximum number of learning times $T$ of LVQ1 was set to the number of instances of training data.

### Experiment 2

In this experiment, the performance of SILVQ when all the instances were learned many times was verified. The experimental procedure was the same as in Experiment 1, except that each model learned each dataset 30 times. The same datasets used in Experiment 1 were used. The models used were SILVQ–RS, SILVQ–LS with $\theta =\text{0.5}$, and existing representative algorithms: generalized LVQ (GLVQ)^54^, generalized relevance LVQ (GRLVQ)^55,56^, and robust soft LVQ (RSLVQ)^57^. The number of prototypes per label of the existing algorithm was set to the value of “number of instances of training data / number of labels / 10,” to prevent the number from being too small. Other parameters were set to be the same as those in the study by Nova and Estévez^48^. Please refer to^48^ for further details regarding the various parameters of comparative LVQ.

### Experiment 3

**Figure 2:**
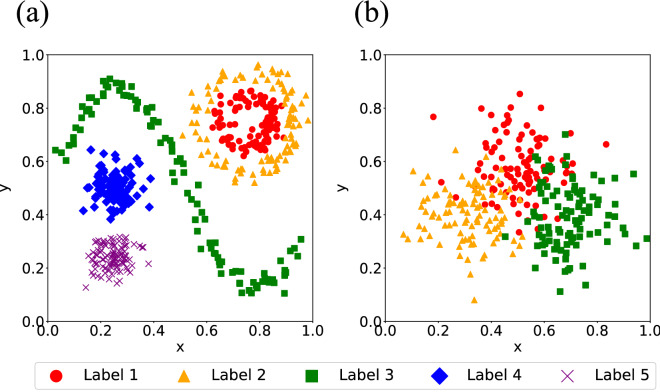
Distribution of artificial datasets used in Experiment 3. (**a**) Distribution of Artificial dataset 1. (**b**) Distribution of Artificial dataset 2.

In this experiment, we verified how a prototype was added when the SILVQ’s confidence threshold $\theta$ was changed. The experimental procedure is the same as in Experiment 2. Two 2-dimensional artificial datasets, Artificial dataset 1 and Artificial dataset 2, were used. Figure 2 shows the distributions of these artificial datasets. Artificial dataset 1 is a non-linearly separable distribution where some labels need to have multiple prototypes for correct classification, whereas Artificial dataset 2 is a distribution where the data for each label are densely overlapping. The model used was SILVQ–LS with $\theta =\text{0.5}$ and $\text{0.8}$. In the case of $\theta =\text{0.5}$, the prototypes are added when the prediction is incorrect even if the label confidence is half (not high). In other words, SILVQ with $\theta =\text{0.5}$ contains several instances as knowledge, similar to EBM. In the case of $\theta =\text{0.8}$, the prototypes are added when the prediction is incorrect and the label confidence is high. This indicates that SILVQ with $\theta =\text{0.8}$ is the SILVQ learning mechanism itself. The initial value of the prototype vector was set as in Experiment 1.

## Results

**Figure 3:**
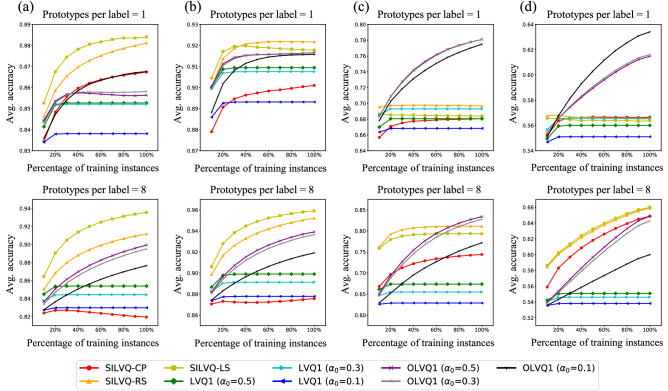
Experiment 1 results. The figure shows the results of 100,000 trials in which each model with prototypes per label = 1 and 8 trained with each dataset 1 time. Each graph shows the average accuracy at each training stage. (**a**) Results for the Glass dataset. (**b**) Results for the Iris dataset. (**c**) Results for the Ionosphere dataset. (**d**) Results for the Sonar dataset.

Figure 3 shows the results from Experiment 1 of 100,000 trials in which each model with prototypes per label = 1 and 8 trained with each dataset 1 time. From the results, SILVQ–RS and SILVQ–LS with prototypes per label = 1 are confirmed to achieve high accuracy by learning a small number of instances, but only for the Glass and Iris datasets. On the other hand, SILVQ–RS and SILVQ–LS with prototypes per label = 8 are confirmed to achieve high accuracy by learning a small number of instances for all datasets.

Table 4 shows the results from Experiment 2 of 100 trials in which each model trained with each dataset 30 times. From the results, SILVQ–LS is confirmed to achieve the same or higher accuracy than that of the existing algorithm, with respect to the median value of accuracy for each dataset.

**Table 4:** Experiment 2 results.

Classifier	SILVQ–RS	SILVQ–LS	GLVQ	GRLVQ	RSLVQ
(a)					
Min	90.7%	**93.0%**	**93.0%**	**93.0%**	81.4%
Max	**100%**	**100%**	**100%**	**100%**	**100%**
Median	97.7%	**100%**	**100%**	**100%**	97.7%
Mean	98.3%	**98.5%**	**98.5%**	**98.5%**	97.8%
Std	0.0204	**0.0176**	0.0179	0.0182	0.0273
(b)					
Min	**86.7%**	**86.7%**	**86.7%**	**86.7%**	80.0%
Max	**100%**	**100%**	**100%**	**100%**	**100%**
Median	**96.7%**	**96.7%**	**96.7%**	**96.7%**	**96.7%**
Mean	95.4%	95.3%	**96.8%**	96.5%	94.7%
Std	0.0364	0.0356	0.0326	**0.0323**	0.0411
(c)					
Min	75.7%	74.3%	**81.4%**	78.6%	75.7%
Max	**95.7%**	**95.7%**	**95.7%**	**95.7%**	**95.7%**
Median	87.1%	**88.6%**	87.1%	87.1%	**88.6%**
Mean	86.7%	87.5%	87.7%	87.5%	**87.7%**
Std	0.0399	0.0403	**0.0297**	0.0357	0.0393
(d)					
Min	59.5%	**71.4%**	50.0%	61.9%	69.0%
Max	**95.2%**	92.9%	88.1%	**95.2%**	**95.2%**
Median	**83.3%**	**83.3%**	66.7%	73.8%	**83.3%**
Mean	82.7%	**83.3%**	68.3%	74.3%	82.4%
Std	0.1070	**0.0510**	0.0753	0.0610	0.0585

The table lists the results of 100 trials in which each model trained with each dataset 30 times. The values in the table include the minimum, maximum, median, mean, and standard deviation of accuracy.

Best results for each dataset are in bold face.

(a) Results for Glass dataset. (b) Results for Iris dataset. (c) Results for Ionosphere dataset. (d) Results for Sonar dataset.

**Figure 4:**
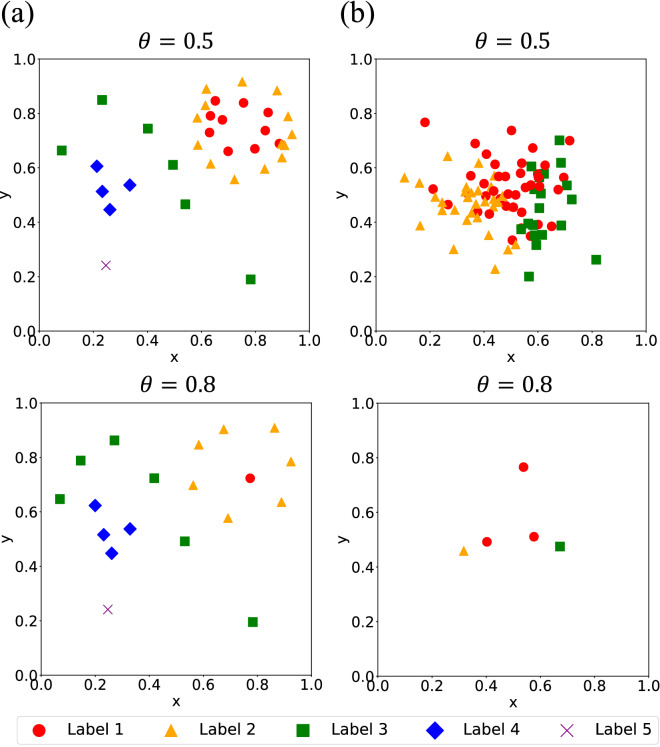
Experiment 3 results. The figure shows results of 1 trial in which SILVQ–LS with confidence threshold $\theta =\text{0.5}$ and $\text{0.8}$ trained with each dataset 30 times. Points in the figure indicate prototypes corresponding to each label. (**a**) Results for Artificial dataset 1. (**b**) Results for Artificial dataset 2.

Figure 4 shows the results from Experiment 3 of 1 trial in which SILVQ–LS with $\theta =\text{0.5}$ and $\text{0.8}$ trained with each dataset 30 times. From the results, all models are confirmed to have appropriately learned Artificial dataset 1; however, SILVQ–LS with $\theta =\text{0.8}$, compared to with $\theta =\text{0.5}$, appropriately learned the instances with a necessary and sufficient number of prototypes. On the other hand, for Artificial dataset 2, SILVQ–LS with $\theta =\text{0.5}$ is confirmed to have overfitted the data. By contrast, SILVQ–LS with $\theta =\text{0.8}$ is confirmed to have learned the instances appropriately with the necessary and sufficient number of prototypes, similar to the result for Artificial dataset 1.

## Discussion

**Figure 5:**
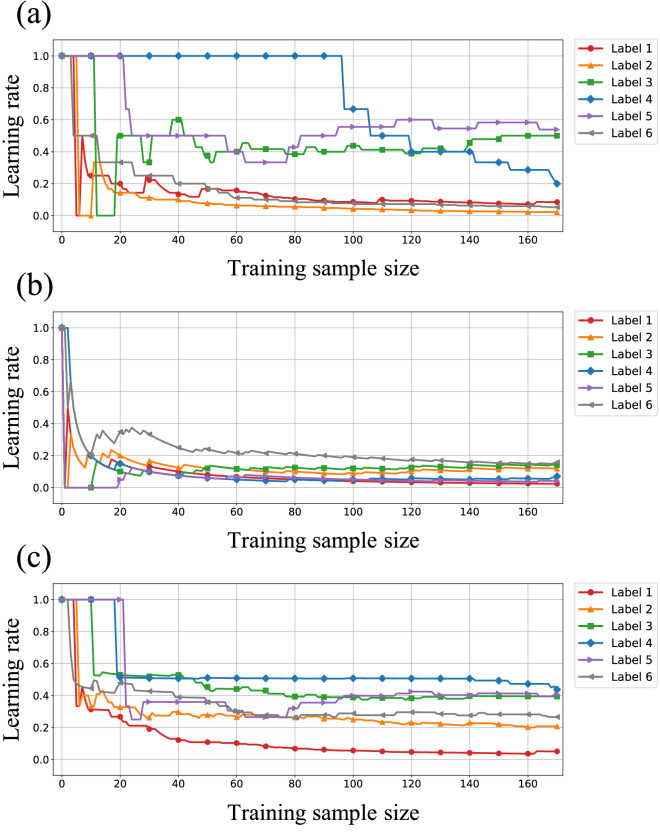
Transition diagram of the learning rate in SILVQ. The figure shows the learning rate of each model at each learning stage of the Glass dataset. Each model has six learning rates corresponding to each label. (**a**) Results for SILVQ–CP. (**b**) Results for SILVQ–RS. (**c**) Results for SILVQ–LS.

The results of Experiment 1 reveal that SILVQ–RS and SILVQ–LS each have a better performance than that of SILVQ–CP. SILVQ–RS and SILVQ–LS include symmetric bias and mutually exclusive bias as human cognitive biases, and update the learning rate heuristically. Therefore, these models are speculated to be good at learning datasets with human-understandable attributes, such as Glass and Iris, but are not good at learning waveform data that are difficult for humans to understand, such as Ionosphere and Sonar. Note that these results are very interesting, but only speculative. However, when learning one instance, SILVQ–CP updates only one label confidence, whereas SILVQ–RS and SILVQ–LS update multiple label confidences. Therefore, SILVQ–RS and SILVQ–LS enable efficient learning from a small number of instances. Figure 5 shows the learning rate of each model at each learning stage of the Glass dataset. When learning an instance, SILVQ–CP only updates the learning rate of the label corresponding to the instance; therefore, it takes time for all learning rates to decrease. In contrast, it can be observed that SILVQ–RS and SILVQ–LS efficiently reduce the learning rate corresponding to all labels. However, SILVQ–RS exhibits strong learning based on the symmetry and mutual exclusivity biases; therefore, the learning process may end early even if it is not performed correctly.

The updating mechanism of these models is based on illogical inferences that derive “Other than this is not an apple” from the teaching “this is an apple.” Most humans have experienced such illogical inferences. For example, you will drive a car based on knowledge learned at a driving school. However, from your experience of good driving, you may implicitly infer “this is good driving” and learn unconsciously that “other than this is bad driving.” Such illogical inference-based learning may not be necessary for machine learning techniques that require perfect performance, but this kind of learning is very human-like.

The results of Experiment 1 also show that, for all datasets, SILVQ–RS and SILVQ–LS can achieve high accuracy with small numbers of instances by increasing the number of prototypes per label. The results of Experiment 2, meanwhile, demonstrate that SILVQ–LS with $\theta =\text{0.5}$ can achieve the same or better accuracy than that of the existing algorithm, without parameters having to be set. However, the purpose of our research is not to develop machine learning models with excellent performance, but to model and elucidate human cognitive processes. Therefore, we want to focus particularly on SILVQ–LS with $\theta =\text{0.8}$, the performance of which is demonstrated by the results of Experiment 3. Most real-world data are complex and noisy. Furthermore, human beings have limited storage capacities and vital energies, and thus cannot store information on all instances in the brain. Therefore, SILVQ–LS with $\theta =\text{0.8}$, which learns two artificial datasets with a necessary and sufficient number of prototypes, can be said to be a very human-like model. Even when the SILVQ learning mechanism is considered, a threshold of 0.8 indicating high confidence would not be qualitatively wrong.

SILVQ can solve some problems related to PBM in a similar manner as EBM by adding instances that characterize each label. In other words, SILVQ can be considered a hybrid model of PBM and EBM. The hybrid model of PBM and EBM is being investigated in the field of cognitive science, such as linguistics; it is not a new idea^58^. However, this discussion is not active in the field of machine learning. This is because artificial intelligence is generally aimed at high-precision learning; therefore, most tasks can be performed like EBM by including or learning a large number of instances. Considering concept formation models in the field of cognitive science may not be necessary in normal machine learning tasks, but it is essential for building human-like artificial intelligence. We hope that our model, which has an easy-to-interpret learning mechanism, will contribute to the fields of both computer science and cognitive science. However, this study does not provide any evidence of similarity between our model and the human cognitive processes; accordingly, further work is required.

## Conclusion

In this paper, we proposed SILVQ as an easy-to-interpret machine learning model incorporating symmetric bias and mutually exclusive bias. The performance of the proposed method was then verified in three experiments using four real and two artificial datasets. SILVQ effectively removed the need for parameter tuning and achieved higher accuracy by learning a small number of samples compared to the original LVQ algorithms. Even when learning a large number of samples, the accuracy of SILVQ was equal to or better than the existing representative LVQ algorithms. Furthermore, SILVQ learned a nonlinearly segregated conceptual structure with the required and sufficient number of prototypes without overfitting.

To improve SILVQ performance, distances other than the Euclidean distance, such as cosine distance, may be used instead. As with advanced LVQ algorithms, such as GLVQ, GRLVQ, and RSLVQ, designing models to strictly minimize classification errors may be possible. However, we challenge ourselves and others not only to improve the performance of our method but also to make it closer to the human cognitive process. Human learning is variable and compound; it is not exclusively based on minimizing the classification errors^59,60^. In future, we will improve our model based on the psychological distance instead of physical distance, such as Euclidean distance, and compare it with human cognitive processes.
